# JMJD1B mediates H4R3me2s reprogramming to maintain DNA demethylation status in neural progenitor cells during embryonic development

**DOI:** 10.1016/j.cellin.2023.100114

**Published:** 2023-08-05

**Authors:** Li Zheng, Huifang Dai, Haitao Sun, Mian Zhou, Eric Zheng, Runxiang Qiu, Qiang Lu, Christina Wei, Binghui Shen

**Affiliations:** Departments of Cancer Genetics and Epigenetics, Beckman Research Institute, City of Hope, 1500 East Duarte Road, Duarte, CA, USA; Departments of Stem Cell Biology and Regenerative Medicine, Beckman Research Institute, City of Hope, 1500 East Duarte Road, Duarte, CA, USA; Departments of Pathology, Beckman Research Institute, City of Hope, 1500 East Duarte Road, Duarte, CA, USA; Departments of Cancer Genetics and Epigenetics, Beckman Research Institute, City of Hope, 1500 East Duarte Road, Duarte, CA, USA

E

*Dear Editor,*

Histone arginine methylation has recently emerged as an important form of histone modifications for regulating gene expression and other cellular processes (Blanc & Richard, 2017). A long-standing hypothesis is that the dynamic status of histone arginine methylation is controlled by the interplay between the family of protein arginine methyltransferases (PRMTs), the “writer”, and the family of protein arginine demethylases, the “eraser” (Blanc & Richard, 2017). While the roles of different PRMTs are relatively well defined, arginine demethylases and their corresponding substrates are largely not identified. We recently demonstrated that JMJD1B (KDM3B), a Jumonji-domain-containing protein, is a methyl-“eraser” that specifically catalyzes the demethylation of H4R3me2s *in vitro* and *in vivo* (Li et al., 2018). We found JMJD1B's demethylase function is crucial for self-renewal, survival, and differentiation in hematopoietic stem cells (Li et al., 2018). A recent study has implicated the germline pathogenic mutations in *JMJD1B* as the cause for intellectual disability, developmental delay, and dysmorphic features including ocular abnormality in 14 unrelated individuals with constitutional syndrome. The genotype-phenotype correlation raises the possibility that *JMJD1B* is involved in the development of the neural-ocular system (Diets et al., 2019). However, the mechanistic link is unclear. Herein, using a *Jmjd1b* homozygous knockout mouse model and immunofluorescence microscopy, we report the novel finding that links JMJD1B as a modulator of global histone arginine methylation level in neural stem/progenitor cells (NSPCs) during embryonic days from E9.5 to E12.5 (brain and ocular development phase). We found that *Jmjd1b* is indispensable for normal ocular development in mice. We observed eye abnormalities (Fig. 1A and B) including small eyes, corneal opacity, corneal-lenticular adhesions, absence of ciliary body and iris development, and failure to open the eye lid in all *Jmjd1b* knockout (*Jmjd1b*^*−/−*^, 129P2 background) mice (2–4 months old, n = 23, Fisher exact test, p < 0.0001) but not in the WT mice (2–4 months, n = 21). This is in keeping with the prior report suggesting that *Jmjd1b* is crucial for neural and ocular development.

**Fig. 1:**
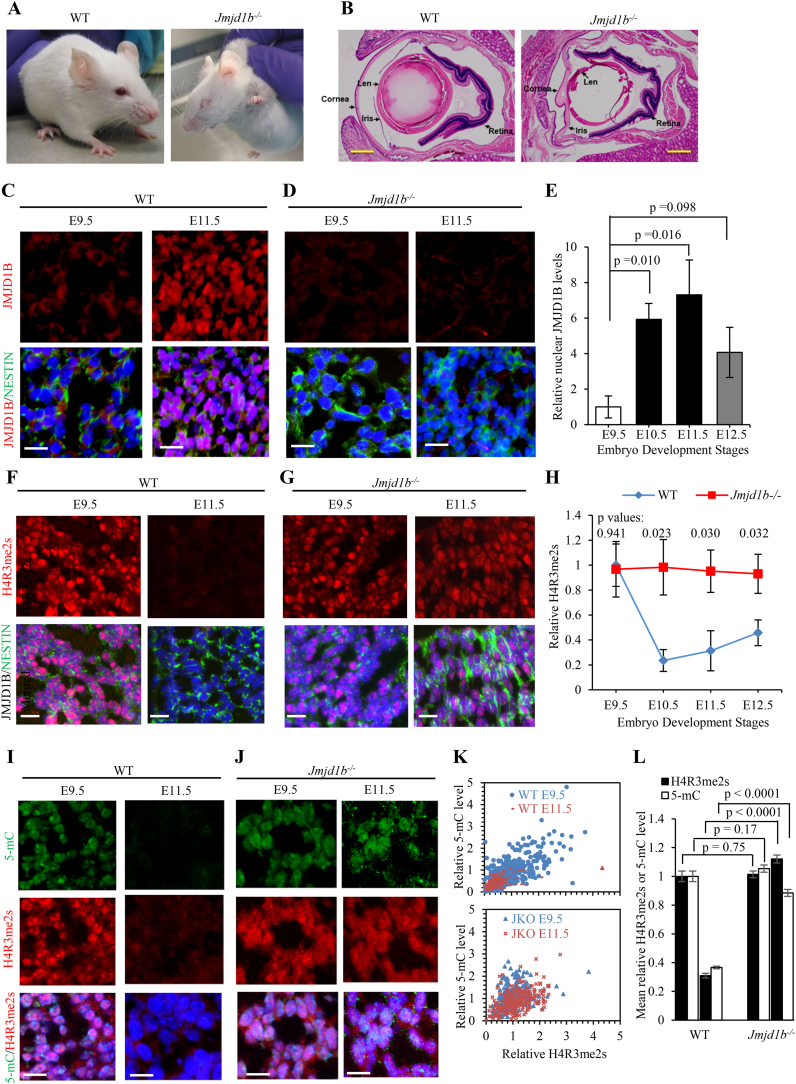
**JMJD1B mediates programmed H4R3 arginine demethylation *in NSPCs*. (A)** Representative images of WT (n = 21) and *Jmjd1b*^*−/−*^ (n = 23) littermates (2–4 months old). **(B)** Histo-pathological analysis of eyes from WT and *Jmjd1b*^*−/−*^ mice (2 months old). The mouse head was dissected and fixed in 10% formalin overnight. The fixed head was sectioned, and H&E staining was conducted to analyze the structural alterations in *Jmjd1b*^*−/−*^. The major structures of the mouse eyes are specified. Scale bars, 500 μm. **(C)–(E)** Levels of JMJD1B in neural progenitor cells. The immunofluorescence co-staining for JMJD1B and NESTIN on whole-embryo tissue sections (cortex areas) of WT (Panel C) and *Jmjd1b*^*−/−*^ (negative control, Panel D) mice at embryonic days 9.5 (E9.5) to12.5 (E12.5). Nuclei were stained with DAPI (blue). Panels C and D show the representative images of JMJD1B (red)/NESTIN (green) co-staining at the cortex area in the WT and *Jmjd1b*^*−/−*^ embryos (E9.5 and E11.5). Scale bars: 40 μm. Panel E is the quantification of nuclear JMJD1B in the NSPCs of different stages. The mean intensity of nuclear JMJD1B staining (red) in the nuclei (blue) of NSPCs (with cytosolic green staining) was quantified with ImageJ. The non-specific staining of JMJD1B (*Jmjd1b*^*−/−*^ littermates) was subtracted from that in the WT. The nuclear JMJD1B level at WT (E9.5) was arbitrarily set as 1 and was used as the reference to calculate the relative nuclear JMJD1B level in WT embryos at other stages. Two male and two female embryos at each stage were stained and five randomly selected views were averaged for each embryo. Values are means ± s. e.m. of the four measurements for each stage. (**F**)**–**(**H**). H4R3me2s levels in NSPCs in WT and *Jmjd1b*^*−/−*^ embryos at E9.5 -E12.5. Panels F and G show the representative micro-images of H4R3me2s in the cortex area of WT or *Jmjd1b*^*−/−*^ embryos (E9.5 and E11.5), respectively. Red: H4R3me2s; green: NESTIN; and blue: nucleus. Scale bars: 40 μm. Panel H shows the quantification of nuclear H4R3me2s levels in WT and *Jmjd1b*^*−/−*^ NSPCs. Values are means ± s. e.m. All p values are calculated by the student's t-test. The H4R3me2s level in WT (E9.5) was arbitrarily set as 1 and was used as the reference to calculate the relative nuclear H4R3me2s levels in WT embryos at other stages. **(I)–(L)** Correlation of H4R3me2s and 5-mC levels in the head sections of WT or *Jmjd1b*^*−/−*^ embryos. Panel I and panel J show representative images of H4R3me2s (red)/5 mC (green) co-staining at the cortex area in the WT and *Jmjd1b*^*−/−*^ embryos (E9.5 and E11.5). Nuclei (blue) are stained with DAPI. Scale bars: 40 μm. Panels K and L show the quantification of nuclear H4R3me2s and 5-mC levels in WT and *Jmjd1b*^*−/−*^ cells in the cortex area. The mean level of nuclear H4R3me2s or 5-mC level at WT (E9.5) was arbitrarily set as 1 and was used as the reference to calculate the relative nuclear H4R3me2s or 5-mC level in WT or *Jmjd1b*^*−/−*^ embryos at different stages. Panel K shows the spread plots (5-mC vs H4R3me2s), which show the correlation of H4R3me2s and 5-mC. Linear regression constants including correlation coefficient (multiple R) and p (significance F) values are calculated using MS-Excel. Panel L shows the mean relative level of H4R3me2s or 5-mC in the WT and *Jmjd1b*^*−/−*^ cells. Values are means ± s. e.m of greater than 200 nuclei in each sample. p values are calculated with the student's t-test.

We studied the dynamic level of JMJD1B protein during neuro-ocular developmental period corresponding to mouse embryonic days from E9.5 to E12.5 and found that nuclear JMJD1B level varies across this developmental period, suggesting a functional role of JMJD1B (Fig. 1C–E). Next, we correlated the global H4R3me2s level in NSPCs, which are defined by expression of NESTIN (NESTIN^+^) (Suzuki et al., 2010) in the cortex and eyes of the embryos. We found that the global H4R3me2s level also varies across neural-ocular developmental period (Fig. 1F–H). Consistent with our hypothesis that JMJD1B modulates H4R3me2s programming, we saw that the global H4R3me2 level is inversely correlated with the expression level of JMJD1B in a temporally coordinated manner. At embryonic day E9.5, NSPCs showed low level of nuclear JMJD1B (Fig. 1C and E) and peak levels of H4R3me2s (Fig. 1F and H) in NSPCs at the cortex area. At E10.5 and E11.5, the JMJD1B levels increased by 7–8 folds (Fig. 1C and E), coincided with a dramatic reduction in the H4R3me2s levels in cortex areas including the eye area (Fig. 1F and H, Supplementary Fig. S1). However, in *Jmjd1b*^*−/−*^ mouse embryos global H4R3me2 did not significantly change from the day E9.5 to E12.5 (Fig. 1G and H). Taken together, in WT mice with normal neural/optical development, we observed a temporal variation in the levels of JMJD1B and H4R3me2 from the day E9.5 to E12.5. In contrast, in *Jmjd1b*^*−/−*^ mice with neural/optical developmental defects, the NSPCs did not show temporal variation in the levels of H4R3me2 from the day E9.5 to E12.5. The expression of H4R3me2 inversely mirrors the directionality of JMJD1B levels. Moreover, our data further supports our hypothesis that JMJD1B's arginine methyl-eraser function is crucial in neuro-ocular development by modulating H4R3me2 level.

Our evidence suggests a regulatory role of JMJD1B-mediated demethylation of H4R3me2s in NSPCs during neural-ocular development. We sought to determine the biological importance of JMJD1B-mediated H4R3me2s demethylation. H4R3me2s was previously suggested as a molecular marker for recruiting DNMT3a to carry out DNA methylation to achieve gene silencing (Zhao et al., 2009). We propose that H4R3me2s demethylation by JMJD1B is important for maintaining DNA demethylation status for gene expression during embryo brain and ocular development. To test this hypothesis, we correlated DNA methylation marker 5-methylcytosine (5-mC) and H4R3me2s in head tissue sections (cortex areas) of the WT and JMHD1B^−/-^ at the day E9.5 and E11.5 (Fig. 1I–L), representing two stages with or without H4R3me2s demethylation, respectively. We observed that the level of H4R3me2s had a significantly positive correlation with the 5-mC level (R = 0.59, p = 1.71 x 10^−33^) in the head section of the WT embryo at E9.5, and H4R3me2s demethylation accompanied with reduction of 5-mC in the head section of the WT embryo at E11.5 (Fig. 1I, K, 1L). However, *Jmjd1b*^*−/−*^ cells had a significantly higher H4R3me2s or 5-mC level than the WT at E11.5 (Fig. 1J, K, 1L), suggesting that *Jmjd1b* deletion disrupts H4R3me2s demethylation and abolished 5-mC reduction at this stage. This finding supports the hypothesis that JMJD1B-mediated H4R3me2s demethylation is an important event for maintaining DNA demethylation status.

We then turned our attention to global H3K9me2 level (JMJD1B's other known demethylase activity), to assess its contribution to neuro-ocular development. In WT mice, at E9.5, we observed little H3K9me2 was detected in the NSPCs at the cortex area, which increased at E10.5 and plateaued at E11.5 (Supplementary Figs. S2A and S2B). As expected, in *Jmjd1b*^*−/−*^ embryos, H3K9me2 level in E9.5 and E10.5 were significantly higher than that in the WT NSPCs (Supplementary Figs. S2A and S2B). However, between the E11.5 and E12.5 stages, global H3K9me2 levels are similar in the NSPCs of both WT and *Jmjd1b*^*−/−*^ embryos (Supplementary Figs. S2A and S2B). This suggests that the drop in H3K9me2 level in *Jmjd1b*^*−/−*^ mice is mediated by the other histone demethylases. On balance, these findings indicate that JMJD1B mediates the global H3K9me2 demethylation early in neuro-ocular development at E9.5 and E10.5 and other methyl-“erasers” may also contribute to H3K9me2 demethylation during other developmental timepoints.

JMJD1B can catalyze both histone lysine and arginine demethylation. Both H4R3me2s and H3K9me2 are suppressive epigenetic markers and are linked to DNA methylation status for suppressing gene expression. However, H4R3me2s and H3K9me2 occur at distinct genomic loci. We previously showed that JMJD1B was crucial for maintaining demethylation status of H4R3me2s or H3K9me2 at the promotor region of distinct genes in hematopoietic stem cells (Li et al., 2018). In current study, we further reveal that JMJD1B-mediated H4R3me2s demethylation occurred at different stages from that for JMJD1B-mediated H3K9me2 demethylation. The sequential JMJD1B-mediated H3K9me2 and H4R3me2s demethylation offers an important mechanism for neural stem cells to program DNA methylation and gene expression for their development differentiation. Because JMJD1B-mediated arginine demethylation activity at H4R3me2s was an independent activity from its histone lysine demethylation activity at H3K9me2, there must be regulatory mechanisms for JMJD1B to achieve such substrate specificity at different development stages. Future studies will define the factors that control the substrate specificity and help us to understand the distinct roles of lysine and arginine demethylation activities of JMJD1B in neural development.

## Declaration of competing interest

The authors have declared no conflict of interest.

## References

[bib1] Blanc R.S., Richard S. (2017). Arginine methylation: The coming of age. Molecular Cell.

[bib2] Diets I.J., van der Donk R., Baltrunaite K., Waanders E., Reijnders M.R.F., Dingemans A.J.M., Pfundt R., Vulto-van Silfhout A.T., Wiel L., Gilissen C. (2019). De novo and inherited pathogenic variants in KDM3B cause intellectual disability, short stature, and facial dysmorphism. The American Journal of Human Genetics.

[bib3] Li S., Ali S., Duan X., Liu S., Du J., Liu C., Dai H., Zhou M., Zhou L., Yang L. (2018). JMJD1B demethylates H4R3me2s and H3K9me2 to facilitate gene expression for development of hematopoietic stem and progenitor cells. Cell Reports.

[bib4] Suzuki S., Namiki J., Shibata S., Mastuzaki Y., Okano H. (2010). The neural stem/progenitor cell marker nestin is expressed in proliferative endothelial cells, but not in mature vasculature. Journal of Histochemistry and Cytochemistry : Official Journal of the Histochemistry Society.

[bib5] Zhao Q., Rank G., Tan Y.T., Li H., Moritz R.L., Simpson R.J., Cerruti L., Curtis D.J., Patel D.J., Allis C.D. (2009). PRMT5-mediated methylation of histone H4R3 recruits DNMT3A, coupling histone and DNA methylation in gene silencing. Nature Structural & Molecular Biology.

