# Supplementary material for: JMJD1B mediates H4R3me2s reprogramming to maintain DNA demethylation status in neural progenitor cells during embryonic development
Source: Cell Insight. 2023 Aug 5;2(4):100114. doi: 10.1016/j.cellin.2023.100114 (PMC10448266; doi:10.1016/j.cellin.2023.100114)
Supplement: Multimedia component 1 [file mmc1.pdf]

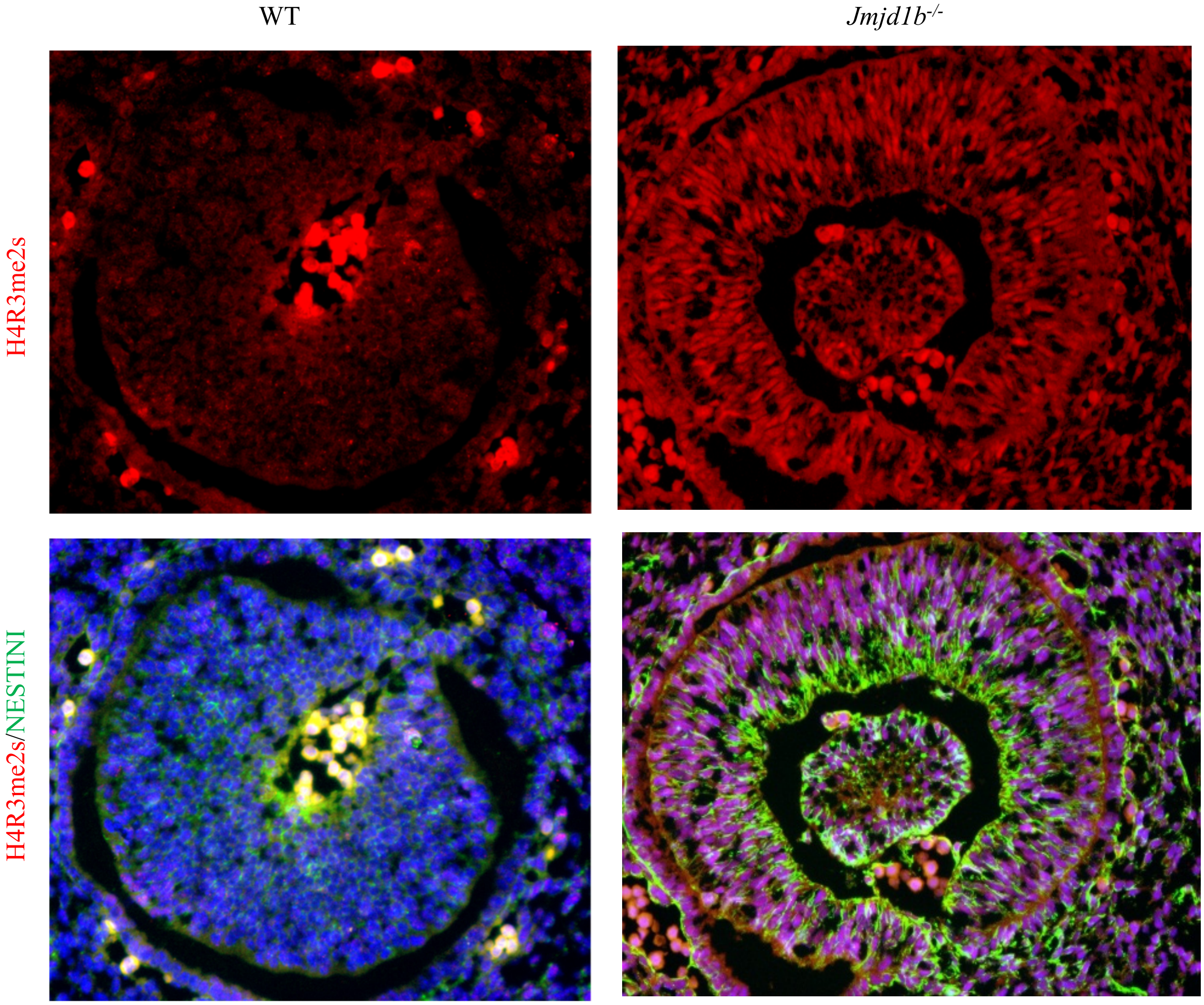

Figure S1. H4R3me2s in NPSC cells in the eye areas of WT and *Jmjd1b*$^{-/-}$ mice. Immuno-fluorescence co-staining of H4R3me2s and NESTIN was conducted on tissue sections (eye areas) of WT and *Jmjd1b*$^{-/-}$ embryos (E10.5). Nuclei were stained with DAPI. Red: H4R3me2s; green: NESTIN, which specifies NSPCs; blue: nuclei.

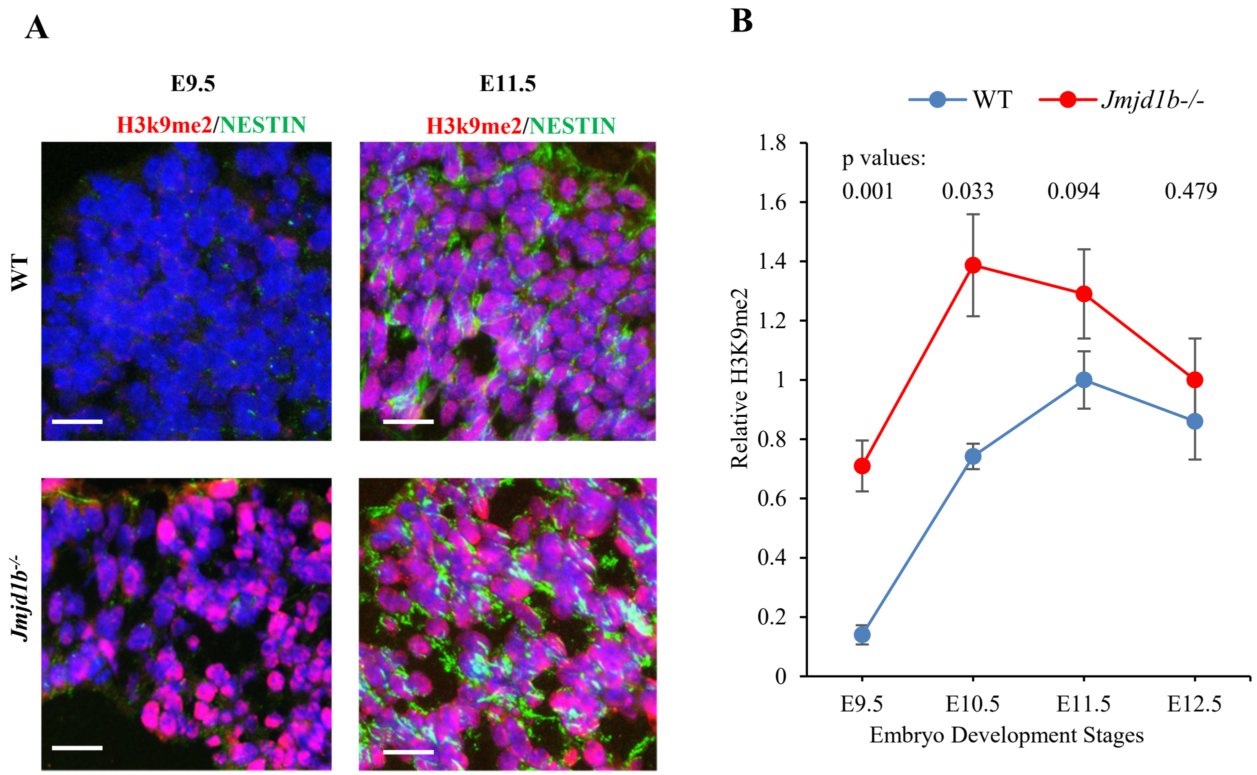

Figure S2. H3K9me2 in NPSCs of WT and *Jmjd1b*$^{-/-}$ embryos. (A) Representative images of immuno-fluorescence co-staining of H3K9me2(Red) and NESTIN (green) was conducted on tissue sections (cortex areas) of WT and *Jmjd1b*$^{-/-}$ embryos (E9.5 and E11.5). Nuclei (blue) were stained with DAPI. **(B)** Quantification of H3K9me2 head sections of WT and *Jmjd1b*$^{-/-}$ embryos by Image J. The H3K9me2 level in WT (E11.5) was arbitrarily set as 1 and was used as the reference to calculate the relative H3K9me2 levels at other stages. p values are calculated by the student's t-test.
